# Low (0–5) Alberta Stroke Program Early Computed Tomography Score on admission predictive of worse functional outcome after mechanical thrombectomy for anterior circulation large vessel occlusion

**DOI:** 10.1186/s40001-023-01225-0

**Published:** 2023-08-04

**Authors:** Jinze Li, Jinfeng Duan, Luojin Zhang, Jingshu Chen, Yang Duan, Benqiang Yang

**Affiliations:** 1 Jinzhou Medical University General Hospital of Northern Theater Command Postgraduate Training Base, Shenyang, China; 2 Center for Neuroimaging, Department of Radiology, General Hospital of Northern Theater Command, Shenyang, China; 3 Department of Surgery, General Hospital of Northern Theater Command, Shenyang, China; 4 Dalian Medical University General Hospital of Northern Theater Command Postgraduate Training Base, Shenyang, China; 5 Department of Radiology, General Hospital of Northern Theater Command, 83 Wenhua Road, Shenhe District, Shenyang, 110016 Liaoning China

**Keywords:** Mechanical thrombectomy, Stroke imaging, Acute ischemic stroke, CT, Outcomes

## Abstract

**Background and purpose:**

We examined functional outcomes of mechanical thrombectomy (MT) procedures following anterior circulation large vessel occlusion (ACLVO)-related acute ischemic strokes (AIS). Results were based on admission non-contrast computed tomography (NCCT) studies, using the Alberta Stroke Program Early Computed Tomography Score (ASPECTS) as standard metric.

**Methods:**

Qualifying subjects were consecutive patients (*N* = 343) at a single center undergoing MT for ACLVO-related AIS. Each was grouped according to ASPECTS status on admission, determined from NCCT images by two physicians. Primary clinical endpoint was functional independence, assessed via modified Rankin Scale (mRS) at 90 days. Secondary endpoints were vessel recanalization (i.e., modified Thrombolysis in Cerebral Infarction [mTICI] score), symptomatic intracranial hemorrhage (sICH), and mortality.

**Results:**

In this study population (mean age, 63.6 ± 12.6 years; women, 30.3%; median baseline National Institute of Health Stroke Scale [NIHSS] score, 15.2 ± 4.5), patients were stratified by ASPECTS tier at presentation, either 0–5 (*n* = 50) or 6–10 (*n* = 293). Multivariate logistic regression showed a relation between ASPECTS values ≤ 5 and lesser chance of 90-day functional improvement (OR = 2.309, 95% confidence interval [CI] 1.012–5.271; *p* = 0.047), once adjusted for age, baseline NIHSS score, diabetes mellitus, HbA1c concentration, D-dimer level, occlusive location, numbers of device passes, and successful recanalization.

**Conclusions:**

ASPECTS values ≤ 5 correspond with worse long-term functional improvement (mRS scores > 2) in patients undergoing MT for ACLVO-related AIS. Other independent determinants of functional outcomes after MT are age, baseline NIHSS score, HbA1c concentration, and successful recanalization.

**Supplementary Information:**

The online version contains supplementary material available at 10.1186/s40001-023-01225-0.

## Introduction

Domestic and foreign studies have validated the Alberta Stroke Program Early Computed Tomography Score (ASPECTS) as a standardized quantitative metric, reflecting the extent of infarction in instances of acute anterior circulation large vessel occlusion (ACLVO) [1, 2]. Recent reports have also demonstrated that this simple and reliable measure may serve to identify the most suitable patients for endovascular therapy and function as a prognostic index [3]. According to American Heart Association/American Stroke Association guidelines, the standard of care in adult patients with ASPECTS values ≥ 6 and large-vessel occlusion is endovascular therapy [4, 5]. However, much of the research on mechanical thrombectomy (MT) has excluded patients with lower ASPECTS values (≤ 6 or 7), failing to fully address the prognostic utility of ASPECTS in MT-treated patients [6, 7]. Only a few of patients with low ASPECTS accepted MT; therefore, data about their clinical outcomes remain scarce. In addition, whether MT is beneficial in AIS patients with low ASPECTS remains uncertain. Hence, in the present study, we compared ASPECTS values of NCCT studies on admission with clinical consequences of MT (including successful recanalization, functional improvement, symptomatic intracranial hemorrhage [sICH], and mortality), hoping to facilitate the planning of MT procedures.

## Methods

### Patient selection

This was a single-center retrospective observational analysis of 343 consecutive patients with ACLVO-related AIS treated by MT between January 2016 and December 2022. The hospital's institutional review board approved our study protocol.

**Fig. 1:**
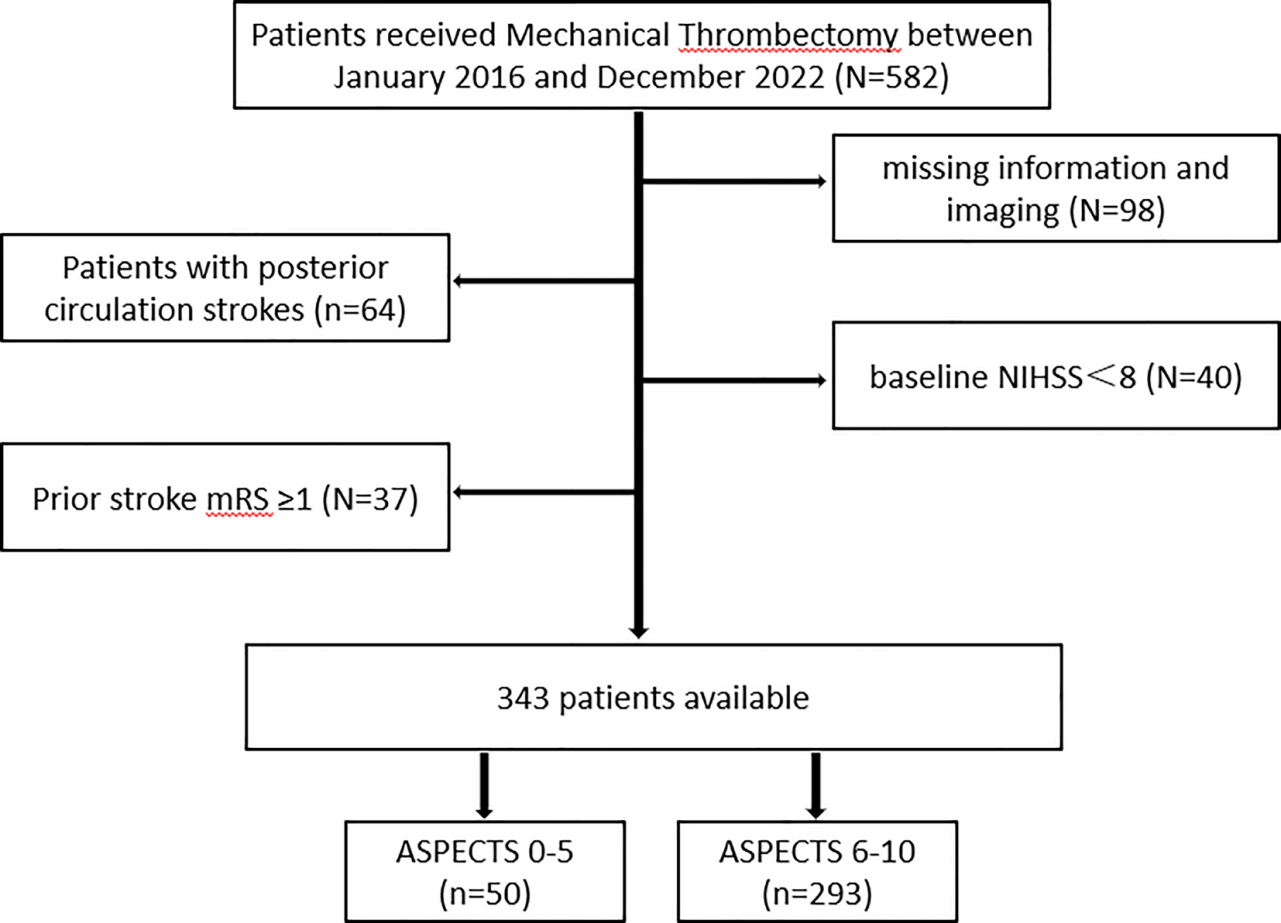
Schematic of patient selection

Eligible MT-treated subjects met the following inclusion criteria: (1) adult ≥ 18 years; (2) availability of non-contrast computed tomography (NCCT) imaging done on admission; and (3) proximal anterior circulation occlusion involving internal carotid artery (ICA) or M1/proximal M2 branch of middle cerebral artery (MCA), as shown by computed tomography angiography (CTA) or digital subtraction angiography (DSA). The following were grounds for study exclusion: (1) prior severe stroke, with baseline National Institutes of Health Stroke Scale (NIHSS) score ≤ 8 or baseline modified Rankin Scale (mRS) score ≥ 1; (2) hemorrhagic infarction on baseline CT; (3) other brain abnormalities, such as tumors or trauma (shown in Fig. 1); or (4) participants with incomplete data.

### Data collection

We retrieved baseline patient characteristics (age and sex), medical history (including prior stroke, coronary artery disease, atrial fibrillation/flutter, hypertension, hyperlipidemia, or diabetes mellitus), and lifestyle habits (current/prior tobacco use, alcohol intake), as well as clinical parameters on admission (ie, NIHSS score, diastolic [DBP] and systolic [SBP] blood pressure readings, glycosylated hemoglobin [HbA1c] concentration, D-dimer level, and presence of hyperdense middle cerebral artery sign [HMCAS] or signs of early infarction on NCCT) and intravenous injection of tissue plasminogen activator (tPA) from electronic medical records. Other clinical variables, including trial of ORG 10172 in acute stroke treatment (TOAST) classification, occlusive location, stroke-onset to puncture time, number of device passes, and use of balloon-guided catheter or angioplasty/stenting, were also recorded.

All NCCT studies were performed by Discovery CT 750 HD scanner (GE Healthcare, Chicago, IL, USA) at the following settings: tube voltage, 100 kV; tube current, 120 mA; collimator width, 40 mm; field of view, 25 cm; layer thickness, 5 mm; layer spacing, 5 mm. Two neuroradiologists blinded to clinical outcomes separately determined ASPECTS values for each patient. Inconsistencies were resolved by imaging reviews, reaching consensus decisions through discussion.

**Fig. 2:**
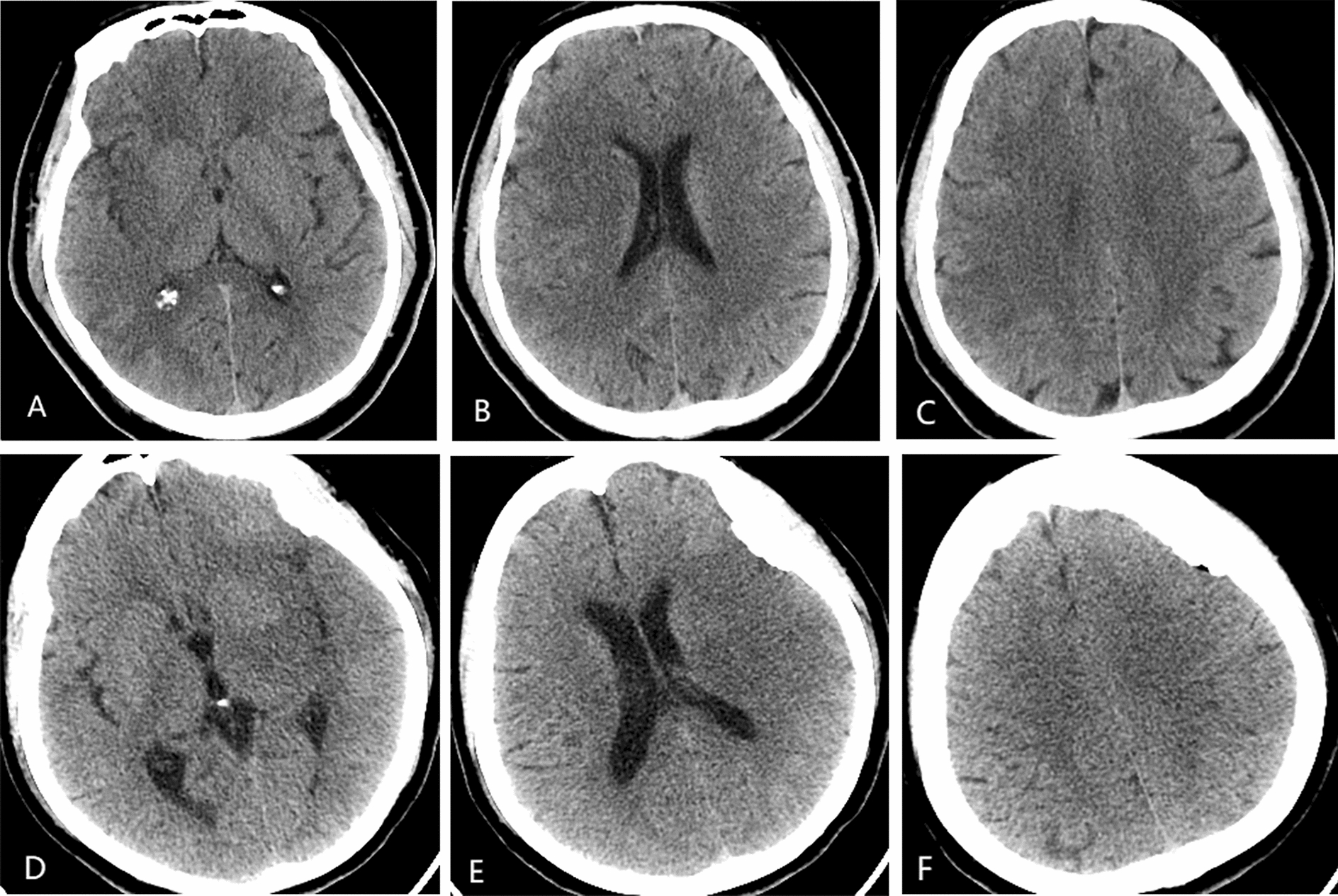
Representative images of patients in ASPECTS tiers: **A**–**C** 56-year-old man with baseline ASPECTS of 6 (M1, M2, L, M6); baseline NIHSS score, 11; HbA1c, 9.22; mTICI score, 2b; and mRS score, 2. **D**–**F** 77-year-old woman with baseline ASPECTS of 4(M2, I, L, IC, M5, M6); baseline NIHSS score, 20; HbA1c, 5.38; mTICI score, 2b; and mRS score, 3. Middle cerebral artery (MCA), insula (I), lenticular nucleus (L), caudate nucleus (C), posterior limb of internal capsule (IC)

In ASPECTS determinations, MCA vascular supply has 10 defined territories [1], including seven regions of caudate nucleus and layers below (M1, M2, M3, insula [I], lenticular nucleus[L], caudate nucleus [C], and posterior limb of internal capsule [IC]) and three areas of cerebral cortex above the nucleus(M4, M5, and M6). Maximum score is 10 points, subtracting 1 point for each injured area (shown in Fig. 2).

### Clinical outcomes

The primary study endpoint was long-term functional independence, measured by mRS at 90 days. Outpatient follow-up or phone calls were used to assess patients functional independence by neurologists. Secondary endpoints were successful vascular reperfusion immediately following MT (ie, modified Thrombolysis in Cerebral Infarction [mTICI] score of 2b or 3)[8]; symptomatic intracranial hemorrhage (sICH), indicated by ≥ 4-point decline in NIHSS score or CT evidence of hemorrhage within 24 h post-MT; and mortality [9].

### Statistical analysis

All computations were driven by standard software (SPSS v22.6; IBM Corp, Armonk, NY, USA). Categorical variables were expressed as frequencies and percentages. The Shapiro–Wilk test was applied to determine normality of distributions. Continuous variables with non-normal distributions were expressed as mean ± standard deviation (SD) or median (interquartile range [IQR]) values. Normally distributed continuous variables were subjected to Student’s *t* test, assesssing non-normal variables by Mann–Whitney *U* test and categorical variables by Fisher’s exact or χ2 test. Multivariate logistic regression analysis served to identify independent predictors of clinical outcomes. Results were presented as odds ratios (ORs) with 95% confidence intervals (CIs), setting significance at *p* < 0.05.

## Results

### Baseline patient characteristics

The 343 qualifying patients were grouped according to ASPECTS tier (0–5, 50; 6–10, 293). Mean age was 63.6 ± 12.6 years, and 96 (30.3%) were women. A summary of group parameters is provided in Table 1. Members of the low-scoring (0–5) group exhibited more extreme presentations than did high-scoring (6–10) group members (mean baseline NIHSS score: 18.1 ± 5.1 vs 14.6 ± 4.3; *p* < 0.001). Likewise, HMCAS positivity (78.0% vs 58.7%; *p* = 0.010) and signs of early infarction (90.0% vs 58.0%; *p* < 0.001) were significantly more prevalent in the low-scoring (vs high-scoring) group (Table 1; Additional file 1: Table S1)

**Table 1:** Baseline patient characteristics, overall and by ASPECTS determinations

Characteristic	Patients, No. (%)			
	Overall (*N* = 343)	ASPECTS0–5 (*n* = 50)	ASPECTS 6–10 (*n* = 293)	*P* value
Age, y	63.6 ± 12.6	61.1 ± 11.0	64.1 ± 12.8	0.058
Female	104(30.3)	9(18)	95(32.4)	**0.044**^a^
Hypertension	198(57.7)	30(60)	168(57.3)	0.725
Diabetes mellitus	90(26.2)	11(22)	79(27)	0.410
Hyperlipidemia	104(30.3)	20(40)	84(28.7)	0.107
Atrial fibrillation/flutter	133(38.3)	20(40)	113(38.6)	0.848
Coronary artery disease	67(19.5)	8(16)	59(20.1)	0.495
Current/prior tobacco use	162(47.2)	27(54)	135(46.1)	0.300
Current/prior alcohol intake	167(48.7)	26(52)	141(48.1)	0.612
TOAST classification				0.678
Large artery atherosclerosis	256(74.6)	36(72)	220(75.1)	
Cardioembolism	79(23)	12(24)	67(22.9)	
Other determined etiology	8(2.3)	2(4)	6(2)	
Prestroke	77(22.4)	11(22)	66(22.5)	0.934
Baseline NIHSS	15.2 ± 4.5	18.1 ± 5.1	14.6 ± 4.3	< **0.001**^a^
HMCAS	211(61.5)	39(78)	172(58)	**0.010**^a^
Signs of early infarction	215(62.7)	45(90)	170(58)	< **0.001**^a^
DBP on admission, mmHg	82.8 ± 15.1	82.4 ± 14.6	82.9 ± 15.2	0.999
SBP on admission, mmHg	140.5 ± 25.1	135.1 ± 27.2	141.4 ± 24.5	0.052
HbA1c, mmol/L	6.3(5.7–8.0)	6.7(5.7–8.0)	6.3(5.7–8.0)	0.354
D-dimer, mmol/L	1.3(0.7–3.1)	1.7(0.9–3.8)	1.3(0.7–2.9)	0.192
Occlusive location				0.378
ICA	91(26.5)	17(34)	74(25.3)	
MCA M1	150(43.7)	21(42)	129(44)	
MCA M2	22(6.4)	1(2)	21(7.2)	
ICA + MCA	80(23.3)	11(22)	69(23.5)	
IV tPA	82(23.9)	7(14)	75(25.6)	0.760
Oneset to puncture, min	412.0(233.5–609.0)	422.5(280.7–545.5)	410.0(227.0–615.0)	0.918
Device passes	1.5(1–2)	1.5(1–2)	1.5(1–2)	0.255
Balloon-guided catheter	80(23.3)	10(20)	70(23.9)	0.548
Angioplasty and stenting	26(7.6)	4(8)	22(7.5)	0.903

Significant values in bold

Data expressed as n (%) or mean ± standard devation values

^a^*ASPECTS* Alberta Stroke Program Early CT Score, *TOAST* Trial of Org 10172 in Acute Stroke Treatment, *NIHSS* National Institutes of Health Stroke Scale, *HMCAS* hyperdense middle cerebral artery sign, *DBP* diastolic blood pressure, *SBP* systolic blood pressure, *HbA1c* glycosylated hemoglobin, *ICA* internal carotid artery, *MCA* middle cerebral artery, *IV tPA* intravenous tissue plasminogen activator

### Clinical outcomes

**Fig. 3:**
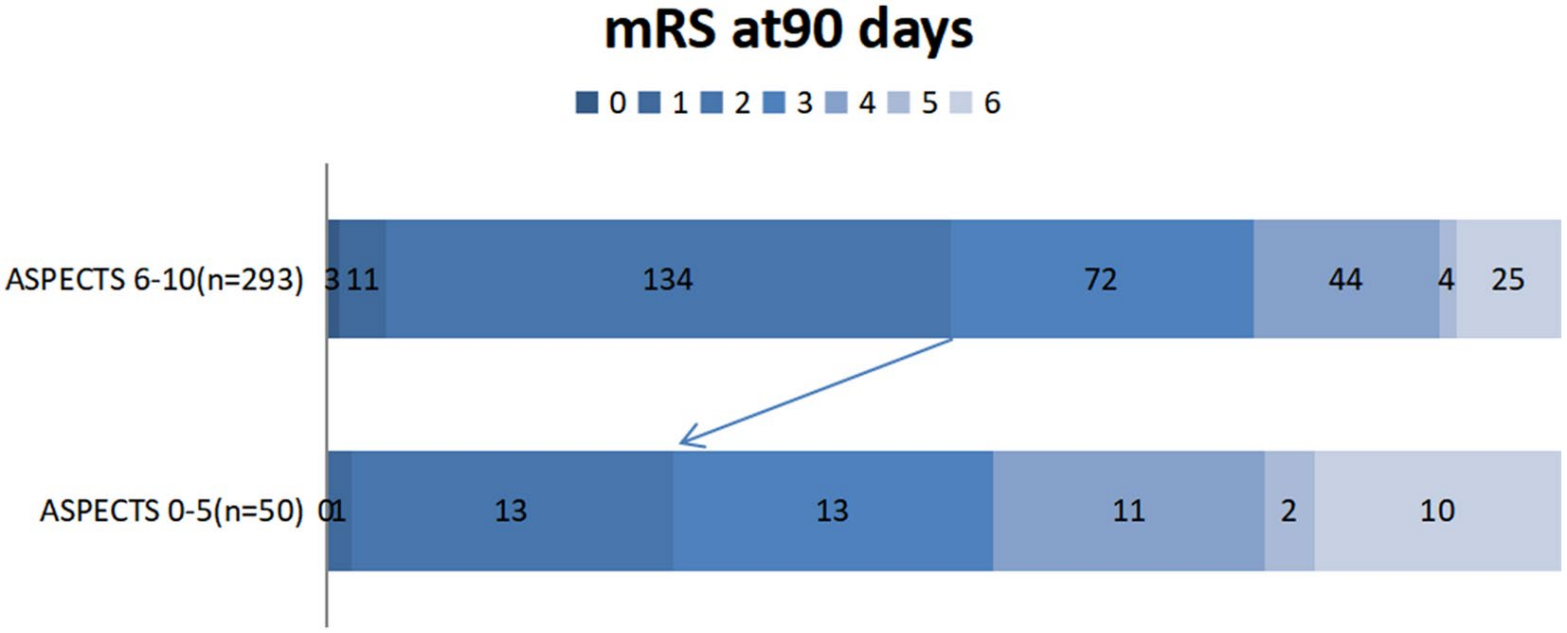
Functional outcomes (modified Rankin Scale [mRS]) at 90 days for two tiers of Alberta Stroke Program Early CT Score (ASPECTS)

Clinical outcomes are shown by groups in Table 2. Unlike the high (6–10) ASPECTS tier, low-tier (0–5) members were less inclined to show good clinical outcomes (mRS scores 0–2) at 90 days (28.0% vs 50.5%; *p* = 0.038) (shown in Fig. 3), demonstrating higher rates of sICH (44.0% vs 21.8%; *p* < 0.001) and mortality (20.0% vs 8.5%; *p* = 0.013). However, successful reperfusion rates did not differ significantly in the two groups (78.0% vs 75.4%; *p* = 0.0695).

**Table 2:** Clinical outcomes of patients, overall and by ASPECTS determinations

Outcomes	Patients, No. (%)			*P* value
	Overall (*N* = 343)	ASPECTS0–5 (*n* = 50)	ASPECTS 6–10 (*n* = 293)	
mTICI score				** < 0.001**^a^
0	25(7.3)	7(14.0)	18(6.1)	
1	25(7.3)	3(6.0)	22(7.5)	
2a	35(10.2)	2(4.0)	32(10.9)	
2b	115(33.5)	32(64.0)	84(28.7)	
3	143(41.7)	6(12.0)	137(46.8)	
Successful reperfusion	259(75.5)	38(76.0)	221(75.4)	0.695
Symptomatic ICH	86(25.1)	22(44.0)	64(21.8)	**0.001**^a^
Good functional outcomes (mRS score < 2) at 90 days	162(47.2)	14(28.0)	148(50.5)	**0.003**^a^
90-day mRS score	3(2–4)	3(2–4.5)	3(2–4)	**0.038**^a^
Mortality	35(10.2)	10(20.0)	25(8.5)	**0.013**^a^

Significant values in bold

Data expressed as n (%) or mean ± standard devation values

^a^*ASPECTS* Alberta Stroke Program Early CT Score, *mTICI* modified Thrombolysis in Cerebral Infarction, *mRS* modified Rankin Scale, *ICH* intracranial hemorrhage

### Predictors of worse functional outcomes (mRS scores > 2)

Table 3 contains a listing of unfavorable prognosticators, including older age (*p* < 0.001), higher baseline NIHSS score (*p* < 0.001), histories of diabetes mellitus (*p* < 0.001), increased HbA1c concentration (*p* < 0.001), elevated D-dimer level (*p* = 0.006), occlusive location (p = 0.002), number of device passes(*p* = 0.001), successful reperfusion (*p* < 0.001), and initial ASPECTS value ≤ 5 (*p* = 0.017). In multivariate logistic regression, a relation between ASPECTS of ≤ 5 and worse functional outcome (mRS score > 2) at 90 days (OR = 2.309, 95% CI 1.012–5.271; *p* = 0.047) emerged, once adjusted for age, NIHSS score, diabetes mellitus, HbA1c concentration, D-dimer level, occlusive location, number of device passes, and successful reperfusion (Table 4).

**Table 3:** Predictors of poor functional outcome at 90 days (univariate ordinal regression)

Characteristic	OR	95%CI	*P* value
Age, y	1.062	1.041–1.084	** < 0.001**^a^
Female	1.30	0.818–2.067	0.267
Hypertension	1.136	0.740–1.745	0.559
Diabetes mellitus	2.574	1.545–4.287	** < 0.001**^a^
Hyperlipidemia	0.790	0.498–1.252	0.315
Atrial fibrillation/flutter	1.531	0.987–2.373	0.057
Coronary artery disease	1.577	0.914–2.720	0.101
Current/prior tobacco use	0.715	0.467–1.094	0.122
Current/prior alcohol intake	0.669	0.437–1.025	0.065
TOAST classification	0.885	0.578–1.356	0.575
Large artery atherosclerosis			
Cardioembolism			
Other determined etiology			
Prestroke	1.484	0.887–2.483	0.133
Baseline NIHSS score	1.143	1.082–1.208	** < 0.001**^a^
HMCAS	0.857	0.554–1.326	0.489
Signs of early infarction	1.634	1.052–2.539	0.029*
DBP on admission	0.988	0.974–1.002	0.090
SBP on admission	0.997	0.988–1.005	0.461
HbA1c	1.486	1.279–1.728	** < 0.001**^a^
D-dimer	1.144	1.040–1.259	**0.006**^a^
Occlusive location	0.731	0.599–0.892	**0.002**^a^
ICA			
MCA M1			
MCA M2			
ICA + MCA			
IV tPA	1.013	0.617–1.665	0.958
Oneset to puncture, min	0.999	0.999–1.000	0.086
Device passes	1.539	1.186–1.997	**0.001**^a^
Balloon-guided catheter	0.783	0.474–1.292	0.338
Angioplasty and stenting	1.807	0.782–4.176	0.166
Successful reperfusion	0.324	0.189–0.555	** < 0.001**^a^
Symptomatic ICH	1.782	1.080–2.941	**0.024**^**a**^
ASPECTS ≤ 5	2.169	1.147–4.100	**0.017**^a^

Significant values in bold

^a^*TOAST* Trial of Org 10172 in Acute Stroke Treatment, *NIHSS* National Institutes of Health Stroke Scale, *HMCAS* hyperdense middle cerebral artery sign; DBP, diastolic blood pressure, *SBP* systolic blood pressure, *HbA1c* glycosylated hemoglobin, *ICA* internal carotid artery, *MCA* middle cerebral artery, *IV tPA* intravenous tissue plasminogen activator, *ASPECTS* Alberta Stroke Program Early CT Score

**Table 4:** Multivariate logistic regression model of poor functional independence (mRS score > 2 at 90d)

Predictor	Odds ratio	95%CI	*P* value
ASPECTS ≤ 5	2.309	1.012–5.271	**0.047**^a^
Age	1.071	1.045–1.098	** < 0.001**^a^
HbA1c	1.391	1.160–1.669	** < 0.001**^a^
Baseline NIHSS	1.094	1.021–1.172	**0.010**^a^
Successful reperfusion	0.313	0.157–0.626	**0.001**^a^

Significant values in bold

^a^*ASPECTS* Alberta Stroke Program Early CT Score, *HbA1c* glycosylated hemoglobin, *NIHSS* National Institutes of Health Stroke Scale

## Discussion

Present findings indicate that ASPECTS values ≤ 5 on admission NCCT studies reduce the likelihood of long-term functional independence and increase the odds of sICH and mortality after MT in patients with ACLVO-related AIS. On the other hand, higher ASPECTS values (6–10) herald significantly better clinical outcomes and carry less risk of death. These associations proved significant in multivariate regression analysis, with other independent predictors emerging as well.

ASPECTS is a simple and convenient, topographic method of semiquantitatively gauging early ischemic core infarct volume and is deemed strongly predictive of clinical outcomes in the setting of AIS [1]. Typically, it is a stipulation for enrollment in various randomized intravascular treatment trials. Because past efforts seemed to restrict the merits of MT to patients with ASPECTS values ≥ 6, subsequent clinical trials have tended to exclude those with lesser scores [6, 7, 10]. Indeed, a recent meta-analysis of such studies undertaken by Phan et al. to explore ASPECTS as a basis for revascularization suitability has demonstrated the prognostic favorability of a higher (vs lower) ASPECTS status in the course of endovascular thrombectomy [10]. However, more and more efforts are showing that patients with lower ASPECTS values may well benefit from MT under certain conditions [11–14]. In a subgroup analysis of the MR CLEAN randomized phase 3 trial, patients with ASPECTS values of 5–7 were regarded as acceptable candidates for endovascular therapy, otherwise weighing potential treatment benefit and cost-effectiveness of endovascular therapy (in conjunction with various influential factors) at values of 0–4 [3]. An analysis from the STRATIS Registry, conducted by Zaidat et al. and aimed at impacts of age and low (0–5) ASPECTS status (ie, large infarcts) on MT outcomes, has also revealed better clinical outcomes and lower risk of death in patients < 65 (vs > 75) years of age [13].

When comparing our two ASPECTS tiers (0–5 vs 6–10), there was no significant difference in rates of successful recanalization (mTICI scores > 2b). Yet, 90-day functional outcomes in low-scoring patients were better if vessel recanalization was achieved. In MT-treated patients with low ASPECTS values (0–5), Kaesmacher et al. have similarly linked successful recanalization to better outcomes and safety profiles [12]. Results of a meta-analysis by Cagnazzo et al. also suggested that patients with ASPECTS values of 0–6 may benefit from MT, with successful recanalization not only heightening the probability of long-term functional improvement but also reducing sICH occurrences. Still, only about one of four patients with ASPECTS values of 4 retained functional independence after MT, and just 14% had favorable functional outcomes at ASPECTS values of 0–3 [11].

Above findings are compatible with ours, rates of functional independence, sICH, and mortality for the two ASPECTS tiers (0–5 vs 6–10) being significantly different. ASPECTS determinations ≤ 5 also strongly signaled worse 90-day clinical outcomes in our multivariable logistic regression model (Table 4). Ultimately, ASPECTS status is a measure of infarct size, an apparent correlate of functional outcomes after endovascular reperfusion therapy [15, 16]. At lower scores, patients simply have less salvageable volumes of brain tissue and larger infarct volumes, both for shadowing functional deficits.

Until now, identifying patients who might benefit most from endovascular therapy after strokes has remained a serious question. According to our data, younger age, lower baseline NIHSS score, lower HbA1c concentration, and successful recanalization are all independent predictors of favorable outcomes after MT, regardless of ASPECTS status. These parameters are also aligned with reported findings in a series of endovascular therapy recipients [13, 17–21].

For a variety of reasons, we did not examine diffusion-weighted imaging (DWI) or CTA studies in this cohort. NCCT remains the most accessible and economical diagnostic tool for emergency use in patients with strokes, and it is often all that time allows. Moreover, symptom severity or existing contraindications may prohibit DWI or CTA scanning procedures.

There are several limitations of this study worth mentioning. First, this was a single-center endeavor, with a relatively sparse sampling of low-scoring patients (ASPECTS 0–5) that may have skewed the results. In addition, all ASPECTS determinations were based entirely on NCCT images; and given the scarcity of patients with the lowest of scores (ASPECTS 0–3), comparative analysis within the lower ASPECTS tier (4–5 vs 0–3) was not feasible. Whether the lowest scoring patients (0–3) actually benefit from MT remains a topic for further study. The findings provided herein must be corroborated as well by multicenter prospective studies that entail other methods of examination.

## Conclusion

ASPECTS values ≤ 5 signal worse long-term functional status (mRS scores > 2) in patients with ACLVO-related AIS undergoing MT. Older age, elevated HbA1c concentration, and higher baseline NIHSS score are other independent risk factors for poor clinical outcomes, whereas successful recanalization is protective of functional independence.

### Supplementary Information

**Additional file 1: Table S1**. Baseline characteristics and clinical outcomes by ASPECTS 3–5 and ASPECTS 6–10.

## Data Availability

The data sets generated and/or analysed during the current study are available from the corresponding author on reasonable request.

## Abbreviations

MT: Mechanical thrombectomy
AIS: Acute ischemic strokes
ACLVO: Anterior circulation large vessel occlusion
NCCT: Non-contrast computed tomography
ASPECTS: Alberta Stroke Program Early Computed Tomography Score
mRS: Modified Rankin Scale
sICH: Symptomatic intracranial hemorrhage
mTICI: Modified Thrombolysis in Cerebral Infarction
NIHSS: National Institute of Health Stroke Scale
ICA: Internal carotid artery
MCA: Middle cerebral artery
CTA: Computed tomography angiography
DSA: Digital subtraction angiography
DBP: Diastolic blood pressure
SBP: Systolic blood pressure
HMCAS: Hyperdense middle cerebral artery sign
tPA: Tissue plasminogen activator
TOAST: Trial of ORG 10172 in acute stroke treatment
