# Supplementary material for: Low (0–5) Alberta Stroke Program Early Computed Tomography Score on admission predictive of worse functional outcome after mechanical thrombectomy for anterior circulation large vessel occlusion
Source: Eur J Med Res. 2023 Aug 4;28:266. doi: 10.1186/s40001-023-01225-0 (PMC10401741; doi:10.1186/s40001-023-01225-0)
Supplement: Supplementary file 1 — Additional file 1: Table S1. Baseline characteristics and clinical outcomes by ASPECTS 3–5 and ASPECTS 6–10. [file 40001_2023_1225_MOESM1_ESM.docx]

**Supplement Table 1.** Baseline characteristics and clinical outcomes by ASPECTS 3-5 and ASPECTS 6-10

|  | **Patients, No. (%)** | | | | | | |  |  |
| --- | --- | --- | --- | --- | --- | --- | --- | --- | --- |
| **Characteristic** | **ASPECTS3-5 (n=36)** | | **ASPECTS 6-10 (n=293)** | | | ***P*-value** | |  |  |
| Age, y | 60.8±11.8 | | 64.0±12.8 | | | 0.089 | |  |  |
| Female | 7(19.4) | | 95(32.4) | | | **0.112*** | |  |  |
| Hypertension | 22(61.1) | | 168(57.3) | | | 0.665 | |  |  |
| Diabetes mellitus | 8(22.2) | | 79(27.0) | | | 0.543 | |  |  |
| Hyperlipidemia | 16(44.4) | | 84(28.7) | | | 0.052 | |  |  |
| Atrial fibrillation/flutter | 16(44.4) | | 113(38.6) | | | 0.495 | |  |  |
| Coronary artery disease | 6(16.7) | | 59(20.1) | | | 0.622 | |  |  |
| Current/prior tobacco use | 19(52.8) | | 141(48.1) | | | 0.598 | |  |  |
| Current/prior alcohol intake | 21(58.3) | | 135(46.1) | | | 0.165 | |  |  |
| TOAST classification |  | |  | | | 0.402 | |  |  |
| Large artery atherosclerosis | 25(69.4) | | 220(75.1) | | |  | |  |  |
| Cardioembolism | 9(25) | | 67(22.9) | | |  | |  |  |
| Other determined etiology | 2(5.6) | | 6(2.0) | | |  | |  |  |
| Prestroke | 8(22.2) | | 66(22.5) | | | 0.967 | |  |  |
| Baseline NIHSS | 17.5±4.1 | | 14.6±4.3 | | | **＜0.001*** | |  |  |
| HMCAS | 28(77.8) | | 172(58.7) | | | **0.027*** | |  |  |
| Signs of early infarction | 33(91.7) | | 170(58) | | | **＜0.001*** | |  |  |
| DBP on admission, mmHg | 80.5±15.1 | | 82.9±15.2 | | |  | |  |  |
| SBP on admission, mmHg | 133.3±21.9 | | 141.4±24.5 | | | 0.050 | |  |  |
| HbA1c, mmol/L | 6.19(5.66-7.98) | | 6.29(5.69-8.08) | | | 0.926 | |  |  |
| D-dimer, mmol/L | 1.68(0.87-3.89) | | 1.34(0.68-2.94) | | | 0.219 | |  |  |
| Occlusive location |  | |  | | | 0.719 | |  |  |
| ICA | 11(30.6) | | dfsa74(25.3) | | |  | |  |  |
| MCA M1 | 15(41.7) | | 129(44) | | |  | |  |  |
| MCA M2 | 1(2.8) | | 21(7.2) | | |  | |  |  |
| ICA+MCA | 9(25) | | 69(23.5) | | |  | |  |  |
| IV tPA | 4(11.1) | | 75(25.6) | | | 0.055 | |  |  |
| Oneset to puncture, min | 556.5(291.2-806.4) | | 410.0 (227.0-615.0) | | | 0.839 | |  |  |
| Device passes | 2(1-3) | | 1(1-2) | | | **0.046*** | |  |  |
| Balloon-guided catheter | 6(16.7) | | 70(23.9) | | | 0.332 | |  |  |
| Angioplasty and stenting | 2(5.6) | | 22(7.5) | | | 0.671 | |  |  |
| mTICI | |  | |  | | | **＜0.001*** | |  |
| 0 | | 4(11.1) | | 18(6.1) | | |  | |  |
| 1 | | 3(8.3) | | 22(7.5) | | |  | |  |
| 2a | | 1(2.8) | | 32(10.9) | | |  | |  |
| 2b | | 24(66.7) | | 84(28.7) | | |  | |  |
| 3 | | 4(11.1) | | 137(46.8) | | |  | |  |
| Successful reperfusion | | 28(77.8) | | 221(75.4) | | | 0.756 | |  |
| Symptomatic ICH | | 16(44.4) | | 64(21.8) | | | **0.003*** | |  |
| Good functional outcomes (mRS score<2) at 90 days | | 12(33.3) | | 148(50.5) | | | **0.049*** | |  |
| 90-day mRS score | | 3(2-4.5) | | 2(2-3) | | | **0.002*** | |  |
| Mortality | | 8(22.2) | | 25(8.5) | **0.010*** | | | | |

Significant values in bold

Data expressed as n (%) or mean ± standard devation values

*ASPECTS, Alberta Stroke Program Early CT Score; TOAST, Trial of Org 10172 in Acute Stroke Treatment; NIHSS, National Institutes of Health Stroke Scale; HMCAS, hyperdense middle cerebral artery sign; DBP, diastolic blood pressure; SBP, systolic blood pressure; HbA1c, glycosylated hemoglobin; ICA, internal carotid artery; MCA, middle cerebral artery; IV tPA, intravenous tissue plasminogen activator; mTICI, modified Thrombolysis in Cerebral Infarction; mRS, modified Rankin Scale; ICH, intracranial hemorrhage
